# Association between Dietary Glycemic Index and Excess Weight in Pregnant Women in the First Trimester of Pregnancy

**DOI:** 10.1055/s-0038-1676096

**Published:** 2018-12-12

**Authors:** Thais Helena de Pontes Ellery, Helena Alves de Carvalho Sampaio, Antônio Augusto Ferreira Carioca, Bruna Yhang da Costa Silva, Júlio Augusto Gurgel Alves, Fabrício Da Silva Costa, Edward Araujo Júnior, Maria Luísa Pereira de Melo

**Affiliations:** 1 Group of Research in Nutrition and Chronic Diseases, Universidade Estadual do Ceará, Fortaleza, CE, Brazil; 2 Department of Nutrition, Universidade de Fortaleza, Fortaleza, Ceará, Brazil; 3 Department of Nutrition, Instituto Federal de Educação, Ciência e Tecnologia do Ceará, Limoeiro do Norte, CE, Brazil; 4 Department of Maternal and Child, Universidade Federal do Ceará, Fortaleza, Ceará, Brazil; 5 Department of Obstetrics and Gynecology, Ribeirão Preto Medical School, Universidade de São Paulo, Ribeirão Preto, SP, Brazil; 6 Department of Obstetrics and Gynecology, Monash University, Faculty of Medicine, Nursing and Health Sciences, Clayton, Victoria, Australia; 7 Department of Obstetrics, Paulista School of Medicine, Universidade Federal de São Paulo, São Paulo, SP, Brazil; 8 Medicine Course, Universidade Municipal de São Caetano do Sul, São Paulo-SP, Brazil

**Keywords:** pregnancy, glycemic index, glycemic load, excess weight, gestação, índice glicêmico, carga glicêmica, excesso de peso

## Abstract

**Objective** To assess the association between dietary glycemic index (GI) and excess weight in pregnant women in the first trimester of pregnancy.

**Methods** A cross-sectional study in a sample of 217 pregnant women was conducted at the maternal-fetal outpatient clinic of the Hospital Geral de Fortaleza, Fortaleza, state of Ceará, Brazil, for routine ultrasound examinations in the period between 11 and 13 weeks + 6 days of gestation. Weight and height were measured and the gestational body mass index (BMI) was calculated. The women were questioned about their usual body weight prior to the gestation, considering the prepregnancy weight. The dietary GI and the glycemic load (GL) of their diets were calculated and split into tertiles. Analysis of variance (ANOVA) or Kruskal-Walls and chi-squared (χ^2^) statistical tests were employed. A crude logistic regression model and a model adjusted for confounding variables known to influence biological outcomes were constructed. A *p*-value < 0.05 was considered significant for all tests employed.

**Results** The sample group presented a high percentage of prepregnancy and gestational overweight (39.7% and 40.1%, respectively). In the tertile with the higher GI value, there was a lower dietary intake of total fibers (*p* = 0.005) and of soluble fibers (*p* = 0.008). In the third tertile, the dietary GI was associated with overweight in pregnant women in the first trimester of gestation, both in the crude model and in the model adjusted for age, total energy intake, and saturated fatty acids. However, this association was not observed in relation to the GL.

**Conclusion** A high dietary GI was associated with excess weight in women in the first trimester of pregnancy.

## Abstract (pt)

**Resumo:**

**Objetivo** Avaliar a associação entre índice glicêmico (IG) dietético e presença de excesso de peso em gestantes no primeiro trimestre de gestação.

**Métodos** Estudo transversal realizado com 217 gestantes atendidas no Ambulatório de Medicina Materno-Fetal do Hospital Geral de Fortaleza, Fortaleza, CE, para realização de exames ultrassonográficos de rotina no período entre 11 e 13 semanas e 6 dias de gestação. Peso e altura foram obtidos para o cálculo do índice de massa corporal (IMC) gestacional. As mulheres foram questionadas quanto ao peso corporal habitual anterior à gestação, considerado o peso pré-gestacional. O IG e a carga glicêmica (CG) das suas dietas foram calculados e divididos em tercis. As associações foram investigadas por análise de variância (ANOVA, na sigla em inglês) ou pelos testes Kruskal-Walls e qui-quadrado (χ^2^).

**Resultados** O grupo tinha alto percentual de excesso de peso pré-gestacional (39,7%) e gestacional (40,1%). Houve menor consumo de fibras totais (*p* = 0,005) e fibras insolúveis (*p* = 0,008) no tercil de maior valor de IG. No terceiro tercil, o IG da dieta foi associado ao excesso de peso das mulheres no primeiro trimestre de gestação, tanto no modelo bruto como no modelo ajustado para idade, consumo total de energia e de ácidos graxos saturados. No entanto, não se observou esta associação em relação à CG.

**Conclusão** O alto IG da dieta consumida foi associado ao excesso de peso das mulheres no primeiro trimestre da gestação.

## Introduction

Gestational weight gain is the focus of several studies,^1^ ^2^ ^3^ ^4^ ^5^ as a result of the worldwide epidemic of obesity^1^ ^2^ ^3^ and its importance in gestational outcomes.^4^ ^5^ Excessive gestational weight gain has been associated with increased risk of large infants for the gestational age (GA), preeclampsia, gestational diabetes, cephalopelvic disproportion, trauma, asphyxia, and perinatal death.^4^ ^5^ Excessive gestational weight gain is associated with postpartum weight retention.^3^

In the last few years, the relevance of the dietary glycemic index (GI) for the development of obesity has been controversially debated.^6^ ^7^ ^8^ The GI is considered an important determinant of fasting glucose tolerance and of postprandial glycemic response.^7^ ^8^ ^9^ Mechanisms linking the habitual consumption of high-GI foods to body composition include reduced satiety signaling, enhanced carbohydrate oxidation, and decreased fat oxidation in response to habitual postprandial glycemia and insulinemia.^8^ ^9^

The GI quantifies the glycemic variations in response to the dietary carbohydrate consumption, and is defined as the area under the glucose response curve after the intake of 50 g of carbohydrates of a test-food, expressed as a percentage response to the same quantity of carbohydrate of a standard food, measured in the same individual.^10^ The glycemic load (GL) is a measure derived from the quantity and quality (GI) of dietary carbohydrates.^11^

High-GI and/or high-GL diets are independently associated with the development and with the progression of chronic diseases, particularly those associated with insulin resistance.^6^ ^11^ The hypothesis that there is an association between overall dietary GI, GL, and disease risk have been inconsistent with this hypothesis.^12^ ^13^

Therefore, given the importance of the diet to the nutritional status and health of pregnant women, the objective of the present study was to evaluate the association of dietary GI and GL during pregnancy with excess weight (overweight and obesity) in women at prepregnancy and during the first trimester of gestation.

## Methods

A cross-sectional study, part of a larger prospective cohort entitled “Prediction of preeclampsia using the triple vascular test” was conducted at the maternal-fetal outpatient clinic of the Hospital Geral de Fortaleza, Fortaleza, state of Ceará, Brazil. The study was approved by the Local Ethics Committee (reference number 050309/09). All of the participants of the present study were informed of the purpose of the study and of all the potential risks involved before signing a written consent form.

The sample comprised 217 pregnant women that underwent routine ultrasound scans at between 11 and 13 weeks + 6 days of gestation. Weight and height were measured during pregnancy using a Marte digital anthropometric scale (Marte Científica, São Paulo, SP, Brazil), with a capacity of 200 kg and 2 m, with a sensitivity of 50 g and of 0.50 cm, respectively. The women were questioned about their usual body weight prior to the gestation, considering the prepregnancy weight. Their body mass index (BMI) was calculated as their weight in kilograms divided by their height in meters squared (kg/m^2^). The prepregnancy BMI was classified according to the World Health Organization (WHO)^14^ criteria as underweight (< 18.5 kg/m^2^), normal (18.5–24.9 kg/m^2^), overweight (25.0–29.9 kg/m^2^) and obese (≥ 30 kg/m^2^). The gestational BMI was classified using the table of Atalah et al.^15^ Overweight or obese women were grouped together into a category named excess weight.

Dietary intake data was collected through interviews that were applied two the 24 hours dietary recall (24hDR) during 2 non-consecutive days, including one weekend day. The pregnant women informed their daily food intake from the previous 24 hours in household measures and, subsequently, we converted them into grams.^16^ Dietary data were input to the DietWin Profissional 2.0 software (dietWin, Porto Alegre, RS, Brazil), which calculated the nutritional composition of the diets, along with the total daily energy intake in kilocalories (Kcal). The nutrients consumed were adjusted for energy using the residual method^17^ and were expressed in g/1,000 Kcal.

Based on the information available for the chemical composition of the diets, the GI was determined using the table of Brand-Miller et al.^18^ ^19^ For foods whose GIs were not listed in the tables, the value was estimated based on foods with similar characteristics and carbohydrate levels. The daily GI was calculated by multiplying the GI of each food (IG*f*) by the proportion of glycemic carbohydrate in the food item (HCOg*f =* HCO*f* – total fiber of food) regarding the amount of daily glycemic carbohydrate, and summing the resultant values (daily GI = ∑ (GI*f* x HCOg*f*) / ∑ HCOg*f)*. The daily GL was determined by adding the glycemic carbohydrate of each food, in grams, multiplied by its individual GI, and dividing it by 100 (daily GL = ∑ (GI*f* x HCOg*f*) / 100).^20^ The daily GI and GL of each of the two recalls were calculated, and an arithmetic mean of daily GI and GL for each individual was obtained. The usual intake of GI and GL was estimated by the multiple source method (MSM) to correct for interpersonal variability.^21^ The mean values adjusted by the MSM were split into tertiles.

In addition, the presence of under-reporting of dietary intake was analyzed. To this end, the basal metabolic rate (BMR) was estimated using the formulas recommended by the Food and Agriculture Organization (FAO)/WHO,^22^ and the total energy intake/BMR ratio was calculated. Values < 1.5, which is the reference cutoff point, were considered indicative of under-reporting.^23^

The values for dietary intake and anthropometric variables were split into the GI tertiles. Analysis of variance (ANOVA) or the Kruskal-Walls and chi-squared (χ^2^) statistical tests were employed. A crude logistic regression model and a model adjusted for confounding variables known to influence biological outcomes were constructed. A *p*-value < 0.05 was considered significant for all statistical tests used. The IBM SPSS for Windows, Version 20.0 (IBM Corp., Armonk, NY, USA) was used for all analyses.

## Results

The majority (77.9%) of the participants of the present study were between 19 and 34 years old, and most patients (67.7%) were married or living with a partner. The distribution of the women according to their reported race was predominantly mixed (71.4%) and white (24.4%). The anthropometric data from the pregnant women are presented in Table 1. The mean prepregnancy BMI of the population studied was 24.5 kg/m^2^ ( ± 4.4). The prepregnancy BMIs showed that a high percentage (39.7%) of the patients had excess weight (overweight and obese). The gestational BMIs (first trimester of gestation) revealed that 40.1% of the women had excess weight. The mean BMI of the population increased to 25.1 kg/m^2^ ( ± 4.4). Weight gain occurred in most of the women (71.9%), with a mean weight increase of 3.1 kg ( ± 2.5).

**Table 1:** Anthropometric assessment at prepregnancy and during the first trimester of pregnancy

Variable	*n* (%)
*Prepregnancy nutritional diagnosis	
Underweight	9 (4.1)
Normal weight	122 (56.2)
Excess weight	86 (39.7)
**Nutritional diagnosis during pregnancy	
Underweight	34 (15.7)
Normal weight	96 (44.2)
Excess weight	87 (40.1)
Total	217 (100.0)

**Source:** *Rasmussen et al (2009)^14^ and **Atalah et al (1997).^15^

The relationship between sociodemographic profile, GA, BMI, and food consumption with the GI tertiles are shown in Table 2. There was a significant difference in the consumption of total (*p* = 0.005) and insoluble fibers (*p* = 0.008), with a lower intake of this nutrient in the highest GI tertile. The GL varied between the three tertiles (*p* = 0.002) without a specific pattern, but following the variation of carbohydrates, even though the latter did not give a difference between the tertiles.

**Table 2:** Distribution of gestational and dietary intake characteristics according to the glycemic index

Variables	GI			*p-value* *
	54.1–57.4 (*n* = 72)	57.5–58.2 (*n* = 73)	58.3–60.5 (*n* = 72)	
Dietary intake				
Energy, Kcal	1,950.7 (553.0)	1,963.7 (629.5)	1,743.6 (682.2)	0.061
Protein, g/1000 Kcal	44.7 (9.9)	41.9 (12.2)	46.1 (12.2)	0.093
Carbohydrate, g/1000 Kcal	128.8 (19.4)	129.1 (20.4)	123.2 (20.9)	0.150
Dietary fiber, g	20.0 (10.5)	19.7 (9.8)	15.1 (6.3)	0.005¥
Soluble fiber, g	6.9 (4.0)	6.4 (3.3)	5.4 (2.7)	0.071
Insoluble fiber, g	9.4 (5.9)	9.7 (6.6)	7.0 (3.7)	0.008¥
Lipids, g/1,000 Kcal	33.8 (6.7)	34.8 (7.3)	35.4 (8.7)	0.435
SFA, g/1,000 Kcal	10.8 (3.2)	9.8 (3.0)	9.8 (3.1)	0.065
PFA, g/1,000 Kcal	6.4 (3.7)	6.6 (3.4)	7.2 (3.7)	0.250¥
MFA, g/1,000 Kcal	8.8 (2.5)	8.6 (3.0)	9.3 (3.4)	0.354
Glycemic load	141.3 (26.1)	143.7 (38.4)	124.5 (32.2)	0.002¥
Energy under-reporting, %	44 (61.1)	49 (67.1)	53 (73.6)	0.279
Socioeconomic profile				
Age, years old	27.6 (6.3)	27.3 (7.2)	26.8 (6.9)	0.284
Marital Status, married (%)	45 (62.5)	55 (75.3)	47 (65.3)	0.311
Nutritional status				
Gestational age, weeks	12.6 (0.9)	12.8 (0.8)	12.6 (0.9)	0.307
Prepregnancy BMI,^1^ kg/m^2^	24.5 (3.7)	25.2 (4.1)	25.0 (4.6)	0.199
Gestational BMI,^2^ kg/m^2^	24.9 (3.2)	25.5 (4.2)	25.8 (4.6)	0.204
Pre-BMI,^1^ excess weight (%)	26 (36.1)	30 (41.1)	39 (54.2)	0.078
^2^ Gestational BMI, excess weight (%)	36 (50.6)	37 (50.7)	48 (66.7)	0.074

Abbreviations: BMI, body mass index; g, grams; GI, glycemic index; MFA, monounsaturated fatty acids; PFA, polyunsaturated fatty acids; SFA, saturated fatty acids.

**Source:**^#^Rasmussen et al (2009)^14^ and ^##^Atalah et al (1997).^15^

* ANOVA Test: *p* < 0.05.

¥ Kruskal-Wallis and χ^2^: *p* < 0.05.

All of the dietary intake variables and gestational characteristics were tested according to the GI and GL tertiles. Logistic regression models were constructed showing that the high GI values in the third tertile were associated with excess weight (overweight and obesity) of the pregnant women in the first trimester in both the crude model and in the model adjusted for age, total energy intake, saturated fatty acids, and under-reporting. This association was not observed for the GL (Table 3).

**Table 3:** Odds ratio of gestacional body mass index according the tertiles of glycemic index and glycemic load adjusted by the multiple source method

	Gestational BMI* (with and without excess weight)		
	Crude model	Model 1**	Model 2***
	OR (95% CI)	OR (95% CI)	OR (95% CI)
GI MSM			
1^st^ tertile (54.1–57.4)	1.00	1.00	1.00
2^nd^ tertile (57.5–58.2)	1.028 (0.536–1.971)	0.992 (0.504–1.955)	0.993 (0.494–1.996)
3^rd^ tertile (58.3–60.5)	2.000 (1.020–3.922)	1.988 (0.981–4.029)	2.204 (1.064–4.567)
* p*-value	0.045	0.059	0.034
GL MSM			
1^st^ tertile (52.9–119.7)	1.00	1.00	1.00
2^nd^ tertile (120.9–146.1)	1.147 (0.592–2.223)	1.216 (0.566–2.611)	1.201 (0.552–2.614)
3^rd^ tertile (148.4–307.0)	0.756 (0.392–1.458)	1.149 (0.446–2.961)	1.645 (0.583–4.644)
* p*-value	0.402	0.764	0.354

Abbreviations: BMI, body mass index; CI, confidence interval; GI, glycemic index; GL, glycemic load; MSM, multiple source method; OR, odds ratio.

*First trimester; **Adjusted for age (tertile) and total energy intake (tertile); *** Adjusted for age (tertile), total energy intake (tertile), saturated fatty acids (tertiles), and under-reporting (yes or no).

## Discussion

The results of the present study revealed a high rate of excess weight (overweight and obesity) before pregnancy, showing the need for continuous monitoring of weight and food consumption.^3^ ^15^ Adequate weight gain and nutrient intake are fundamental for the gestational period, preventing complications in pregnancy outcomes.^4^ ^5^ ^24^ Prepregnancy excess weight is a risk factor for overweight and obesity during pregnancy. Pregnant women gain weight during this period but beginning the pregnancy with excess weight can lead to an increase in body mass that can affect negatively the health of both the mother and the newborn.^2^ ^25^

Excess weight in the first trimester of pregnancy was found in 40.1% of the women. This group may have included women with excess weight before pregnancy that continued to gain weight. Excessive gestational weight gain has been associated with an increased risk of large infants for the GA,^24^ ^26^ preeclampsia, gestational diabetes, cephalopelvic disproportion, trauma, asphyxia, and perinatal death.^4^ ^5^

Excessive weight gain can lead to an increased risk of postpartum weight retention, influencing a potential obesity that may persist or worsen during the lifetime of the woman.^1^ ^27^ Mattar et al^1^ observed that ∼ 50% of the overweight or obese women had a higher than recommended weight gain, and that > 70% of them maintained the excessive weight up to 12 months postpartum, and 30% had a retention of ≥ 10 kg.

Our results showed a greater risk of overweight in individuals who consumed diets with a higher GI. Sampaio et al^6^ observed the consumption of foods with a high or moderate GI in 78.7% of an obese group, highlighting a high percentage of individuals who consumed diets with inadequate GI at breakfast (82.9%), at afternoon snacks (60.0%), and at dinner (64.6%).

The GI quantifies the glycemic variations in response to the dietary carbohydrate consumption. When diets with high GI are consumed, a glycemic increase occurs due to the high level of glucose, leading to hyperinsulinemia.^28^ Different sources of carbohydrates have varying absorption rates, and their effects on plasma concentrations of glucose and insulin vary accordingly.^29^ ^30^ In the present study, both the intake of total fiber as well as of the insoluble fiber declined by the tertile. Diets containing a higher level of fiber retard the absorption of glucose by the organism, avoiding a rapid increase in blood glucose and reducing the release of insulin by the pancreas.^7^ ^30^

Postprandial glycemia is modulated mainly by the speed of release of carbohydrates derived from the diet into the bloodstream after meals, by the clearance time of the carbohydrates through insulin secretion, and by peripheral tissue sensitivity to the action of this hormone.^20^ ^29^ Thus, the type and amount of dietary carbohydrates are key factors that influence the glycemic response.^30^ ^31^ Studies have shown that pregnant women receiving advice and encouragement to consume a low-GI diet have longer gestational periods,^32^ ^33^ as well as fewer preterm births, although no effects of these diets on infant birth weight have been found in groups at risk of macrosomia.^33^

Another aspect to consider is the GL, which is calculated by multiplying the GI of foods by their glycemic carbohydrate content and reflects directly the quantity and quality of dietary carbohydrates.^7^ ^10^ The GL is one of the most representative characteristics of the overall diet because it indicates the dietary fiber intake.^9^ ^10^ No difference was observed among the GL values in the GI tertiles, probably because the GL quantifies the total effect of a given amount of carbohydrate on plasma glucose, representing the GI product of a food by its available carbohydrate content.^7^ The GL can provide a better reflection of the glycemic response of a specific food than the GI. The glycemic effect of foods varies with the composition of the food and with the methods of preparation.^7^ ^20^ In addition, under-reporting was found in 53% of the pregnant women, which directly impacts the GL values. Usually, women with excess weight tend to not fully disclose their food intake for several reasons, including fear of exposing their poor eating habits.^34^

The level of GI necessary to affect body composition remains unclear. Further elucidation of the mechanisms associated with the potential benefits of consuming carbohydrates, as measured by GI values, is essential before introducing this as a strategy for controlling obesity and its comorbidities, particularly during pregnancy, when weight gain can be expected. Moses et al^32^ found no significant differences in fetal and obstetric outcomes between subjects who followed a low-GI diet versus a higher-GI diet. A randomized controlled trial^35^ reported no difference in birth weight of newborns of mothers consuming a low-GL diet, whereas the gestational period was 10 days longer in the same group, suggesting that this type of diet may be an important factor for preventing prematurity. A meta-analysis that assessed 7 maternal and 11 newborn outcomes observed that low-GI diets may have beneficial effects on maternal outcomes for those at risk of developing high glucose levels, without causing adverse effects on newborn outcomes.^36^

## Conclusion

Based in the results of the present study, it can be concluded that high-GI diets were associated with excess weight in pregnant women in the first trimester of gestation. Therefore, individualized nutritional consultations are recommended in this group to promote dietary improvements.

## Notes

**Erratum:** The ORCID ID of DOI: 10.1055/s-0038-1676096 has been changed from https://orcid.org/0000-0001-7510-0485 to https://orcid.org/0000-0002-6145-2532.
