# Erratum – Association between Dietary Glycemic Index and Excess Weight in Pregnant Women in the First Trimester of Pregnancy

**DOI:** 10.1055/s-0039-1685226

**Published:** 2019-04-16

**Authors:** Thais Helena de Pontes Ellery, Helena Alves de Carvalho Sampaio, Antônio Augusto Ferreira Carioca, Bruna Yhang da Costa Silva, Júlio Augusto Gurgel Alves, Fabrício Da Silva Costa, Edward Araujo Júnior, Maria Luísa Pereira de Melo

**Affiliations:** 1 Group of Research in Nutrition and Chronic Diseases, Universidade Estadual do Ceará, Fortaleza, CE, Brazil; 2 Department of Nutrition, Universidade de Fortaleza, Fortaleza, Ceará, Brazil; 3 Department of Nutrition, Instituto Federal de Educação, Ciência e Tecnologia do Ceará, Limoeiro do Norte, CE, Brazil; 4 Department of Maternal and Child, Universidade Federal do Ceará, Fortaleza, Ceará, Brazil; 5 Department of Obstetrics and Gynecology, Ribeirão Preto Medical School, Universidade de São Paulo, Ribeirão Preto, SP, Brazil; 6 Department of Obstetrics and Gynecology, Monash University, Faculty of Medicine, Nursing and Health Sciences, Clayton, Victoria, Australia; 7 Department of Obstetrics, Paulista School ofMedicine, Universidade Federal de São Paulo, São Paulo, SP, Brazil; 8 Medicine Course, Universidade Municipal de São Caetano do Sul, São Paulo-SP, Brazil

Rio de Janeiro, March 20, 2019

Dear readers,

In the Article *Association between Dietary Glycemic Index and Excess Weight in Pregnant Women in the First Trimester of Pregnancy* (*Rev Bras Ginecol Obstet 2019; 41(01): 04–10 DOI:* 10.1055/s-0038-1676096.), published online in *Rev Bras Ginecol Obstet* in December 2018, where it reads:

ORCID ID is https://orcid.org/0000-0001-7510-0485.

It should read:

Edward Araujo Júnior’s ORCID is https://orcid.org/0000-0002-6145-2532.

Rio de Janeiro, 20 de Março de 2019

Prezados leitores,

No artigo *Associação entre o índice glicêmico dietético e o excesso de peso em gestantes no primeiro trimestre de gestação* (*Rev Bras Ginecol Obstet 2019; 41(01): 04–10 DOI: 10.1055/s-0038-1676096.*), publicado online em *Rev Bras Ginecol Obstet* em Dezembro de 2018, onde se lê:

ORCID ID is https://orcid.org/0000-0001-7510-0485.

Lê-se:

Edward Araujo Júnior's ORCID is https://orcid.org/0000-0002-6145-2532

